# A Novel Role of Hyaluronan and Its Membrane Receptors, CD44 and RHAMM, in Obesity-Related Kidney Pathology

**DOI:** 10.3390/biom15111598

**Published:** 2025-11-14

**Authors:** Bingxue Qi, Vishal Musale, Xiong Weng, Ayman K. Banah, Alexander Lawlor, Colin E. Murdoch, Abigail C. Lay, Kate J. Heesom, Richard J. M. Coward, Christopher L. O’Connor, Wenjun Ju, Markus Bitzer, Claire E. Hills, Yang Chen, Li Kang

**Affiliations:** 1 Division of Diabetes, Endocrinology and Reproductive Biology, School of Medicine, University of Dundee, Dundee DD1 9SY, UKxweng@ed.ac.uk (X.W.); akbanah@uqu.edu.sa (A.K.B.); 2606609@dundee.ac.uk (A.L.); l.kang@dundee.ac.uk (L.K.); 2 Precision Molecular Medicine Center, Jilin Province People’s Hospital, Changchun 130021, China; 3 Division of Cardiovascular Research, School of Medicine, University of Dundee, Dundee DD1 4HN, UK; c.z.murdoch@dundee.ac.uk; 4 Division of Cardiovascular Sciences, University of Manchester, Manchester M13 9PL, UK; abigail.lay@manchester.ac.uk; 5 Bristol Renal, Bristol Medical School, University of Bristol, Bristol BS8 1QU, UK; k.heesom@bristol.ac.uk (K.J.H.); richard.coward@bristol.ac.uk (R.J.M.C.); 6 Division of Nephrology, Department of Internal Medicine, University of Michigan, Ann Arbor, MI 48109, USA; oconnoc@med.umich.edu (C.L.O.); wenjunj@med.umich.edu (W.J.); markusbi@med.umich.edu (M.B.); 7 School of Life Sciences, University of Lincoln, Brayford Pool, Lincoln LN6 7TS, UK; chills@lincoln.ac.uk; 8 Clinical Medicine College, Changchun University of Chinese Medicine, Changchun 130117, China

**Keywords:** hyaluronan, CD44, RHAMM, mice, obesity, insulin resistance, kidney

## Abstract

Obesity-related kidney pathology (ORKP) is a major global issue that contributes to diabetic nephropathy and kidney cancer and leads to chronic/end-stage kidney disease. Current treatments for ORKP are limited because of the incomplete understanding of the disease pathogenesis. Here, we identified a novel role for hyaluronan (HA) and its membrane receptors, CD44 and RHAMM, in this condition. Obesity-induced increases in HA deposition and CD44 and RHAMM expression are detrimental to the kidney via activation of the TGF-β1/Smad2/3, P38/JNK MAPK, and ROCK/ERK pathways, leading to glomerulopathy, tubular injury, inflammation, albuminuria, and elevated serum creatinine concentrations. Either pharmacological or genetic ablation of HA, CD44, or RHAMM reverses these obesity-driven pathologies in vivo. We further established a mechanistic link between renal insulin resistance and ECM remodelling using human kidney cells in vitro, providing insight into the cell type-specific role of HA, CD44, and RHAMM in the pathogenesis of ORKP. Finally, analysis of glomerular and tubular fractions of human kidney biopsy samples revealed increased expression of CD44 and RHAMM in chronic kidney disease and diabetic nephropathy, and their expression correlated with markers of kidney dysfunction. Our findings provide evidence for HA-CD44/RHAMM as a potential therapeutic target in ORKP and subsequent prevention of chronic kidney disease. While previous studies have implicated CD44 and RHAMM in renal disease and fibrosis, our work for the first time provides an integrated analysis of both receptors in the context of ORKP.

## 1. Introduction

The incidence of obesity-related kidney pathology (ORKP) is rising rapidly worldwide, paralleling the global obesity epidemic. ORKP is associated with chronic kidney disease (CKD), end-stage renal disease, and higher mortality rates. Current treatments for ORKP (e.g., weight loss and RAAS inhibitors) are limited and attenuate over time because of the incomplete understanding of the disease pathogenesis. Therefore, understanding the mechanisms of ORKP and developing new early interventions could have significant clinical and socioeconomic benefits.

Hyaluronan (HA), a non-sulphated glycosaminoglycan, is a major extracellular matrix (ECM) constituent and participates in tissue repair and disease progression [1]. In a healthy kidney, HA is mainly located in the interstitium of the inner medulla and is not prominently expressed in the glomerulus [2]. However, glomerular HA increases in response to injury and disease. Under pathophysiological conditions of kidney diseases, such as acute kidney injury (AKI), diabetic nephropathy, lupus nephritis, chronic cyclosporine nephropathy, and IgA nephropathy, elevated contents of HA in the glomerulus are associated with renal inflammation and interstitial fibrosis [3].

CD44 is a major cell surface receptor for HA. Upon HA binding, CD44 interacts with and recruits intracellular signalling molecules to activate the NF-κB, PKC, PI3K/Akt, Src-ERK, and MAPK-signalling pathways [4]. This activation induces inflammatory processes and regulates cell adhesion, proliferation, migration, tumour cell invasion, and cell metabolism [4]. In normal kidneys, CD44 is predominantly expressed in tubular structures including the basolateral membranes of collecting ducts in the inner stripes of outer medulla, the thin descending limb of Henle’s loop, and macula densa cells [5]. However, CD44 can be expressed in the glomerulus, although usually in pathological states. Glomerular CD44 expression was elevated with renal injury, inflammation, and fibrosis in mice [6]. Genetic deletion of CD44 in mice reduces the number of glomerular lesions in crescentic glomerulonephritis [7].

Receptor for HA mediated motility (RHAMM) is another important HA receptor. RHAMM expression is low in homeostatic adult tissues but transiently elevated in response to tissue injury [8]. Although RHAMM differs from CD44 in structure, they appear to perform similar or synergistic functions in many diseases [9]. Evidence suggests that CD44-mediated cellular processes including inflammation, wound healing, tumour formation, and cell migration also require RHAMM surface expression [10]. In contrast to the many studies on CD44 and kidney diseases [7,11], there are few studies on RHAMM, limited to renal cell carcinomas [12]. Recently, we reported that plasma and urine RHAMM expression was negatively associated with eGFR in patients with diabetic kidney disease [13].

We have previously shown that HA accumulation contributes to obesity-associated insulin resistance and that a reduction in HA or the genetic deletion of CD44 improves insulin resistance in obese mice [14,15]. However, the role of HA and CD44 in regulating kidney function in the setting of obesity or ORKP has not been studied. Moreover, the involvement of RHAMM in this process is unknown. In this study, we employed both genetic and pharmacological approaches to reduce the HA-CD44/RHAMM pathway in mice for renal metabolic and functional phenotyping. Together with human kidney cell lines and patient biopsies, our results reveal a novel role of the HA-CD44/RHAMM pathway in the pathogenesis of ORKP.

## 2. Research Design and Methods

### 2.1. Animal Experiments

Animal work was conducted in accordance with the United Kingdom Animals (Scientific Procedures) Act 1986, the ARRIVE guidelines, and the University of Dundee Welfare and Ethical Use of Animals Committee. All mice were housed in an air-conditioned room at 22 ± 2 °C with a 12 h light/dark cycle and had free access to water and food. All mice used in these experiments were male and were fed either a high fat (HF) diet (60% calories as fat, SDS #824054, Merck KGaA, Darmstadt, Germany) or a chow control diet (13% calories as fat, DBM #D/811004, Midland, MI, USA) starting at 6 weeks of age for 16 weeks before being culled for analyses. Considering sex as a biological variable, our study exclusively studied male mice due to their robust response to HF diet-induced obesity and kidney morphological changes; therefore, the current study may limit its clinical relevance only to the male gender.

*Cd44*-null mice (*Cd44*^−/−^) were obtained from Jackson Laboratory ( no. 005085, Bar Harbor, ME, USA). *Hmmr*-null mice (*Hmmr*^−/−^) (encodes RHAMM protein) were a kind gift from Dr Eva Turley (University of Western Ontario) and generated as previously described [16]. For the reduction in the HA content, mice received injections of either vehicle (10 mmol/L histidine, 130 mmol/L NaCl at pH 6.5) or PEGylated recombinant human hyaluronidase PH20 (PEGPH20; provided under a Material Transfer Agreement with Halozyme Therapeutics, San Diego, CA, USA) at 1 mg/kg through the tail vein, once every 3 days for 28 days. The dose and treatment regime of PEGPH20 in this study were chosen based on previous findings demonstrating beneficial effects on insulin sensitivity without adverse effects on appetite or physical activity [14].

To examine the role of HA and its receptors in ORKP in vivo, three mouse studies were conducted, each including 4 experimental groups. **Study 1:** (1) Normal chow diet-fed C57BL/6 mice (Chow); (2) HF diet-fed C57BL/6 mice (HF); (3) HF diet-fed mice receiving vehicle injections (HF Vehicle); (4) HF diet-fed mice receiving PEGPH20 injections (HF PEGPH20). **Study 2:** (1) HF diet-fed CD44 wildtype littermate control mice (HF *Cd44*^+/+^); (2) HF diet-fed *Cd44*-null mice (HF *Cd44*^−/−^); (3) HF diet-fed *Cd44*-null mice with vehicle injections (HF *Cd44*^−/−^ Vehicle); (4) HF diet-fed *Cd44*-null mice with PEGPH20 injections (HF *Cd44*^−/−^ PEGPH20). **Study 3:** (1) Chow diet-fed RHAMM wildtype littermate control mice (Chow *Hmmr*^+/+^); (2) Chow diet-fed *Hmmr*-null mice (Chow *Hmmr*^−/−^); (3) HF diet-fed wildtype control mice (HF *Hmmr*^+/+^); (4) HF diet-fed *Hmmr*-null mice (HF *Hmmr*^−/−^). At the time of sacrifice, blood and kidney tissue were collected. For each mouse, one kidney was snap-frozen at −80 °C for gene and protein expression, and the other was fixed in 10% formalin for histology.

### 2.2. Renal Function Measurement

The serum creatinine concentration was determined using a Creatinine Assay kit (#ab65340, Abcam, Cambridge, UK). Urine albumin concentration was measured using a Mouse Albumin ELISA Kit (#ab108792, Abcam, Cambridge, UK).

### 2.3. Histology and Immunohistochemistry

Paraffin-embedded kidney sections (5 μm) were stained with Periodic Acid–Schiff (PAS) (#ab150680, Abcam, Cambridge, UK), Sirius red (#365548, Sigma, St. Louis, MO, USA), and Picric acid (#P6744, Sigma, St. Louis, MO, USA). Immunohistochemical staining for α-SMA was performed using anti-α-SMA (#D4K9N, Cell Signaling, Danvers, MA, USA, 1:200). HA was assessed using a biotinylated HA-binding protein (#AMS.HKD-BC41, Amsbio, Abingdon, UK, 1:200). Staining and image analysis were conducted in a blinded manner, with ten images of distinct areas per animal captured using an AxioVision microscope (Zeiss, Carl Zeiss AG, Oberkochen, Germany). Sirius red, α-SMA, and HA staining were quantified using ImageJ 1.53k. For PAS, glomerular areas were measured in six random outer cortex glomeruli per section. Tubular damage was scored based on epithelial cell vacuolar deformation/hypertrophy, dilation, brush border loss, and lysis [17].

### 2.4. RNA Extraction and qRT-PCR

Total RNA was isolated from mouse kidneys using TRIzol reagent (#20130301, Ambion, Austin, TX, USA) and reversely transcribed into cDNA (#2409783, Invitrogen, Carlsbad, CA, USA). The mRNA expression was determined by qRT-PCR using an Applied Biosystems (QuantStudio 7 Flex, Thermofisher, Waltham, MA, USA). The sequences of the specific primers are listed in Supplemental Table S1. Data were normalised to the 18S expression and quantified using the 2^−ΔΔCT^ method.

### 2.5. Western Blotting

Whole kidney homogenates were prepared in protein lysis buffer containing 25 mM Tris-HCI pH 7.4, 1% Triton X-100, 50 mM NaF, 0.1 mM NaCI, 1 mM EDTA, 5 mM EGTA, 9.2% sucrose, 10 mM NaPp, 0.1% mercaptoethanol, 1 mM Na_3_VO_4_, 1 mM benzamidine, 0.1 M PMSF, and 10% glycerol using 0.5 mm zirconium oxide beads (#E-1626, ZROB05; Next Advance, Troy, NY, USA). Proteins (40 μg) were separated on 10% SDS-PAGE gels and transferred to nitrocellulose membranes. Protein expression was measured by immunoblotting using primary antibodies (1:1000) specific to CD44, RHAMM, p-Smad2(Ser^465/467^)/Smad3(Ser^423/425^), Smad2/3, p-p38(Thr^180^/Tyr^182^), p38, p-SAPK/JNK(Thr^183^/Tyr^185^), SAPK/JNK, p-Akt(Ser^473^), Akt, p-Erk1/2(Thr^202^/Tyr^204^), Erk1/2, ROCK2 (Cell Signaling, Danvers, MA, USA: #E7k2y, #E7S4Y, #8828S, #3102S, #9211, #9212, #9251, #9252, #4060, #9272, #4370, #4695, #8236, respectively), and TGF-β1 (#ab179695, Abcam, Cambridge, UK). GAPDH (#5174, Cell Signaling, Danvers, MA, USA) or Tubulin (#ab6046, Abcam, Cambridge, UK) served as loading controls.

### 2.6. Cell Culture and Induction of Insulin Resistance

Conditionally immortalised human podocytes [18] and mesangial cells [19] were cultured in RPMI-1640 with L-glutamine, NaHCO_3_, and 10% FBS. Proximal tubular (PT) cells [20] were maintained in DMEM-HAM F-12 with 36 ng/mL hydrocortisone, 10 ng/mL EGF, 40 pg/mL Triiodothyronine, and 10% FBS. Glomerular Endothelial Cells (GEnC) [21] were cultured in Endothelial Cell Growth Medium-2 with microvascular supplements and 5% FBS. Cells were differentiated for 12–14 days at 37 °C and were Mycoplasma-free. To induce an obesogenic and insulin-resistant condition, cells were treated with 100 nmol/L insulin, 25 mmol/L glucose, 1 ng/mL TNF-α, and 1 ng/mL IL-6, with D-Mannitol only as an osmotic ‘Basal’ control [22].

### 2.7. Tandem Mass Tag (TMT)-Mass Spectrometry (MS) Processing and Analysis

The TMT-MS method, including sample processing and data analysis, has been recently published [23]. Briefly, proteins from cells were extracted using RIPA buffer, digested with trypsin, and labelled with TMT reagents (ThermoFisher, Waltham, MA, USA). Labelled samples were pooled, desalted using a SepPak cartridge (Waters, Milford, MA, USA), and fractionated by high pH reversed-phase chromatography on an XBridge C18 column (Waters, Milford, MA, USA) using an Ultimate 3000 system (ThermoFisher, Waltham, MA, USA). Fractions were analysed by nano-LC-MS/MS on an Orbitrap Fusion Lumos mass spectrometer (ThermoFisher) with SPS-MS3 acquisition. Data were processed with Proteome Discoverer v2.1 (ThermoFisher, Waltham, MA, USA), searched against the UniProt human database using SEQUEST, and filtered for a 5% false discovery rate (FDR).

The data output from the Proteome Discoverer 2.1 analysis were normalised with the TMM (trimmed mean of M-values) method and transformed to log2 with the voom method using the limma R package. Different linear models were built on the transformed data on either the ‘Basal’ or ‘Insulin-Resistant’ condition of each independent cell line with five replicates. Expression of each differentially regulated protein was normalised to the ‘Basal’ condition in GEnC cells.

### 2.8. Human Tissue Procurement and Analysis

Patients undergoing total nephrectomy were enrolled in the PRECISE cohort at the University of Michigan, with institutional review board (IRB) approval (HUM00165536). Fresh normal kidney tissue was obtained from the unaffected part of the removed kidney, and patient data were collected from electronic medical records. FFPE tissue blocks were cut at 3 μm and stained with PAS, Masson Trichrome, and Wilms Tumor 1 (#ab89901, Abcam). Slides were scanned at 40× using a Leica AT2 scanner and analysed with ImageJ as previously described [24]. RNALater-preserved tissue was micro-dissected to separate glomeruli and tubulointerstitial fractions [25]. RNA was extracted for gene expression analysis using Illumina (San Diego, CA, USA) sequencing on a NovaSeq X flow cell. Differential gene expression was analysed as previously described [26]. Pearson correlations were performed for statistical analysis with *p* measuring statistical significance, *r* measuring the linear correlation between two variables, and *r*^2^ measuring how close the data were to the fitted regression line.

### 2.9. NephroSeq Analysis

CD44 and RHAMM gene expression was analysed by Nephroseq v5 (https://nephroseq.org/ (accessed on 10 November 2023)) using datasets of Nakagawa CKD Kidney (GSE66494) [27] and Ju CKD Glomeruli and Tubulointerstitium (GSE69438) [28], with the analysis type of ‘Disease vs. Control Analyses’. Correlation of CD44 and RHAMM gene expression with GFR, serum creatinine concentration, and proteinuria were analysed in datasets of Ju CKD Tubulointerstitium and Schmid Diabetes Tubulointerstitium (GSE21785) [29], with the analysis type of ‘Clinical Biomarker Analyses’. Pearson correlations were performed for statistical analysis as described above, with *r* measuring the linear correlation between two variables and *r*^2^ measuring how close the data were to the fitted regression line. Nephroseq analysis did not adjust for age or sex.

### 2.10. Statistics

All data except Nephroseq and correlation analyses were expressed as mean ± SEM and analysed using Prism GraphPad 10. The unpaired two-tailed Student’s *t* test was used to identify statistical significance between two groups. Comparisons among multiple groups were performed using one-way ANOVA followed by Tukey’s multiple comparisons test. The data were considered statistically significant at *p* < 0.05.

## 3. Results

### 3.1. Reduction in HA Attenuated Obesity-Induced Tubular Damage, Renal Dysfunction, and Fibrosis

**Figure 1:**
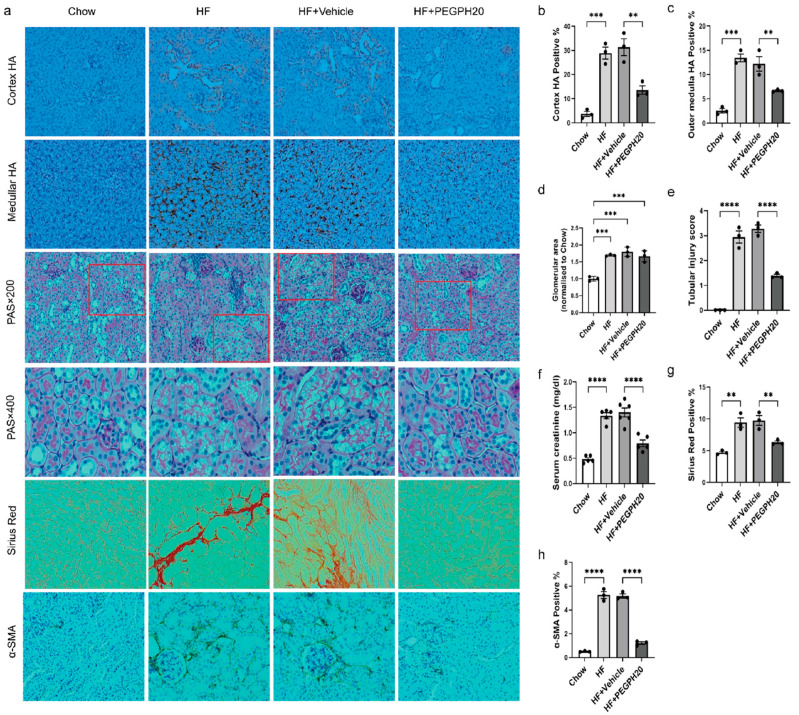
PEGPH20 treatment attenuated obesity-induced HA accumulation, tubular damage, renal dysfunction, and fibrosis. (**a**) Images of HA, PAS, Sirius Red, and α-SMA staining in kidneys from chow-fed mice, HF-fed mice, and HF-fed mice with either vehicle or PEGPH20 (1 mg/kg) treatment. Representative images (×20 magnification) are shown for HA, Sirius Red, and α-SMA staining, and images (×20 and ×40 magnification) are shown for PAS staining. Areas of HA-positive (%), glomeruli, Sirius red-positive (%), and α-SMA-positive (%) were measured using the Image J program. (**b**,**c**) Quantification of HA content. (**d**) Glomerular area. (**e**) Tubular injury score. (**f**) Serum creatinine concentration. (**g**) Quantification of collagen deposition. (**h**) Quantification of α-SMA expression. Data are presented as mean ± SEM. For all panels, one-way ANOVA followed by Tukey’s post-test for multiple comparisons was used for the analysis of statistical significance. Significance: ** *p* < 0.01, *** *p* < 0.001, **** *p* < 0.0001. Areas highlighted by red squares are further magnified to 400×.

HF diet feeding in mice increased HA deposition in the cortex and outer medulla of the kidney, and these increments were partially reversed by PEGPH20 (Figure 1a–c). The glomerular area was increased in the HF diet-fed mice relative to chow-fed controls, which was not affected by PEGPH20 treatment (Figure 1a,d). Furthermore, tubular damage, including tubular epithelial cell vacuolar deformation/hypertrophy, tubular dilation, loss of brush border, and cell lysis, was observed in the cortex and outer stripe of the outer medulla of the kidney in HF diet-fed mice (Figure 1a), which was partially prevented by PEGPH20 (Figure 1e). Serum creatinine concentration, a marker of renal function was increased by HF diet feeding but significantly reversed by PEGPH20 treatment (Figure 1f). Renal fibrosis, assessed by collagen deposition and protein expression of α-SMA was increased in HF-fed mice relative to chow-fed controls, which was also partially reversed by PEGPH20 treatment (Figure 1a,g,h).

### 3.2. PEGPH20 Blocked Inflammation and the Activation of TGF-β1/Smad2/3, P38/JNK MAPK, and HA/CD44 Pathways in Obese Mice

**Figure 2:**
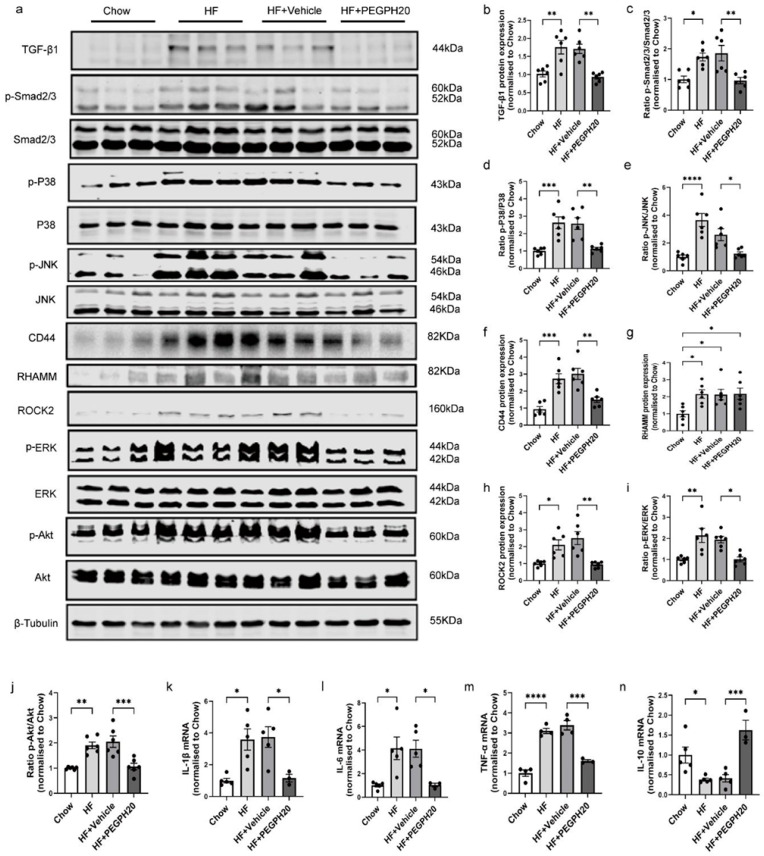
PEGPH20 blocked the activation of the TGF-β1/Smad2/3, P38/JNK MAPK, and HA/CD44 pathways and prevented obesity-induced inflammation. (**a**–**j**) Representative File S1 Western Blot images and quantification of protein expression of the TGF-β1/Smad2/3 pathway, P38/JNK MAPK pathway, and CD44/RHAMM and HA/CD44 pathway-associated proteins (ROCK2/ERK/Akt) in kidneys from chow-fed mice, HF-fed mice, and HF-fed mice with either vehicle or PEGPH20 (1 mg/kg) treatment. N = 6 male mice. (**k**–**n**) mRNA expression of IL-1β (**k**), IL-6 (**l**), TNF-α (**m**), and IL-10 (**n**) assessed by qRT–PCR. N = 3–5 male mice. Data are presented as mean ± SEM. For all panels, one-way ANOVA followed by Tukey’s post-test for multiple comparisons was used for the analysis of statistical significance. Significance: * *p* < 0.05, ** *p* < 0.01, *** *p* < 0.001, **** *p* < 0.0001.

We further investigated the mechanisms by which PEGPH20 ameliorated ORKP. In the whole kidney lysates, TGF-β1/Smad2/3 signalling was increased by HF diet feeding in mice, and this was abolished by PEGPH20 treatment (Figure 2a–c). Moreover, the phosphorylation of P38/JNK MAPK was increased in the HF diet-fed mice, which was blocked in mice treated with PEGPH20 (Figure 2a,d). We next investigated whether the renal protective effect of PEGPH20 was dependent upon CD44 and RHAMM. HF diet feeding increased CD44 and RHAMM expression, and PEGPH20 treatment reduced CD44 (Figure 2a,f) but did not affect RHAMM expression in the kidney of HF-fed mice (Figure 2a,g). Furthermore, HF diet feeding resulted in an increase in ROCK2 expression and ERK and Akt phosphorylation, which were abolished by PEGPH20 (Figure 2a,h–j). ROCK2, ERK, and Akt are important intracellular signalling molecules of HA-CD44 signalling [4]. The HF diet also increased mRNA expression of the pro-inflammatory cytokines IL-1β, TNF-α, and IL-6 and decreased mRNA expression of the anti-inflammatory cytokine IL-10 (Figure 2k–n). These changes were also reversed by PEGPH20.

### 3.3. Global Cd44 Gene Deletion Attenuated Obesity-Induced HA Accumulation, Tubular Damage, Renal Dysfunction, and Fibrosis

**Figure 3:**
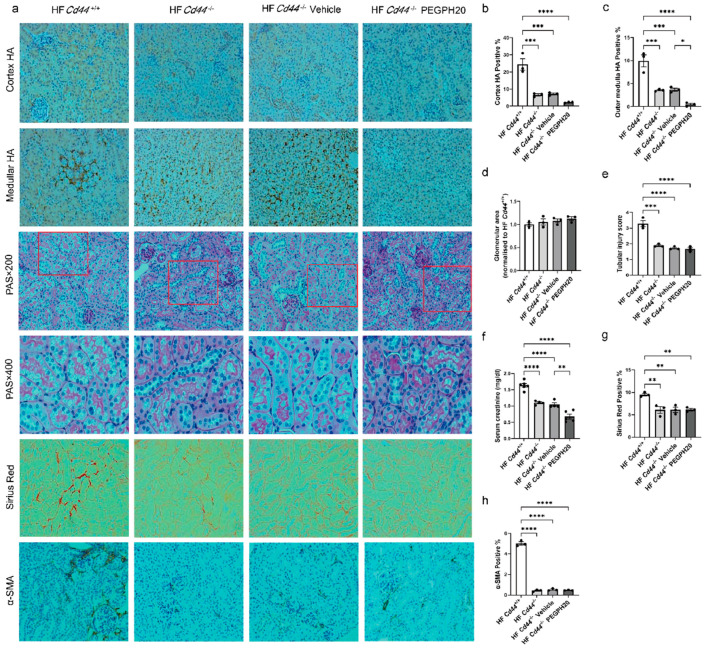
Global *Cd44* gene deletion attenuated obesity-induced HA accumulation, tubular damage, renal dysfunction, and fibrosis. (**a**) Images of HA, PAS, Sirius Red, and α-SMA staining in kidneys from HF-fed *Cd44*^+/+^ mice, HF-fed *Cd44*^−/−^ mice, and HF-fed *Cd44*^−/−^ mice with either vehicle or PEGPH20 (1 mg/kg) treatment. Representative images (×20 magnification) are shown for HA, Sirius Red, and α-SMA staining, and images (×20 and ×40 magnification) are shown for PAS staining. Areas of HA-positive (%), glomeruli, Sirius red-positive (%) and α-SMA-positive (%) were measured using the Image J program. (**b**,**c**) Quantification of HA content. (**d**) Glomerular area. (**e**) Tubular injury score. (**f**) Serum creatinine concentration. (**g**) Quantification of collagen deposition. (**h**) Quantification of α-SMA expression. Data are presented as mean ± SEM. For all panels, one-way ANOVA followed by Tukey’s post-test for multiple comparisons was used for the analysis of statistical significance. Significance: * *p* < 0.05, ** *p* < 0.01, *** *p* < 0.001, **** *p* < 0.0001. Areas highlighted by red squares are further magnified to 400×.

We next elucidated whether CD44 mediated renal injury in ORKP. Global deletion of *Cd44* in HF-fed mice significantly decreased the renal cortex and outer medulla HA accumulation (Figure 3a–c). PEGPH20 treatment in *Cd44*^−/−^ mice caused a further reduction in HA deposition in the outer medulla but not in the cortex (*p* = 0.2508). *Cd44* deletion in HF-fed mice significantly decreased the tubular injury scores (Figure 3a,e), serum creatinine concentrations (Figure 3f), collagen deposition (Figure 3a,g), and α-SMA expression (Figure 3a,h), without affecting the glomerular areas (Figure 3a,d). PEGPH20 treatment caused a further reduction in the serum creatinine concentration in HF-fed *Cd44*^−/−^ mice (Figure 3f).

### 3.4. Global Cd44 Gene Deletion Blocked Inflammation and the Activation of TGF-β1/Smad2/3, P38/JNK MAPK, and HA/CD44 Pathways in Obesity

**Figure 4:**
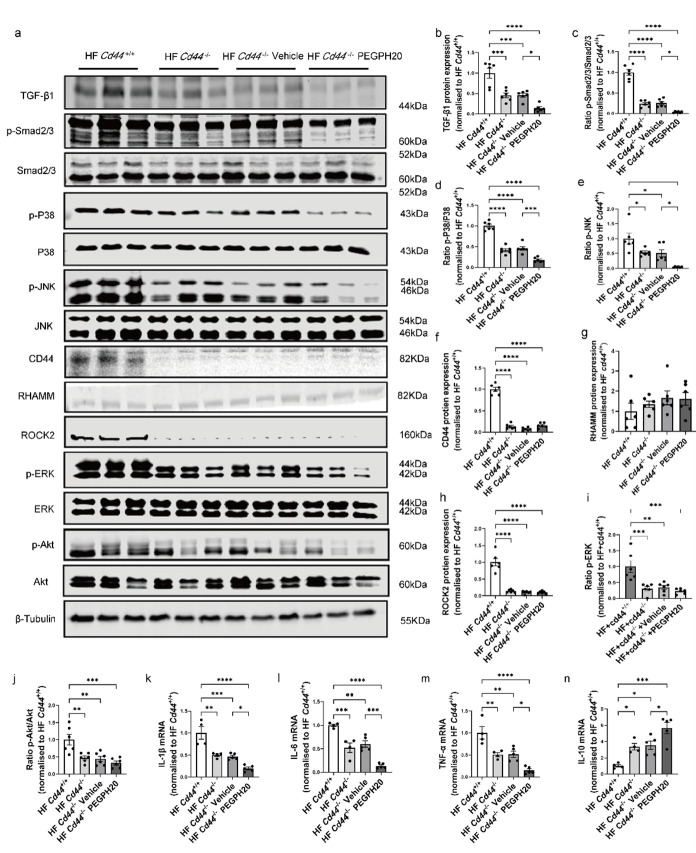
Global *Cd44* gene deletion blocked the activation of TGF-β1/Smad2/3, P38/JNK MAPK, and HA/CD44 pathways and prevented obesity-induced inflammation. (**a**–**j**) Representative File S1 Western Blot images and quantification of protein expression of the TGF-β1/Smad2/3 pathway, P38/JNK MAPK pathway, and CD44/RHAMM and HA/CD44 pathway-associated proteins (ROCK2/ERK/Akt) in kidneys from HF-fed *Cd44*^+/+^ mice, HF-fed *Cd44*^−/−^ mice, and HF-fed *Cd44*^−/−^ mice with either vehicle or PEGPH20 (1 mg/kg) treatment. N = 6 male mice. (**k**–**n**) mRNA expression of IL-1β (**k**), IL-6 (**l**), TNF-α (**m**), and IL-10 (**n**) assessed by qRT–PCR. N = 3–5 male mice. Data are presented as mean ± SEM. For all panels, one-way ANOVA followed by Tukey’s post-test for multiple comparisons was used for the analysis of statistical significance. Significance: * *p* < 0.05, ** *p* < 0.01, *** *p* < 0.001, **** *p* < 0.0001.

Mechanistically, the protein expression of TGF-β1 and phosphorylation of Smad2/3 were reduced in HF-fed *Cd44*^−/−^ mice relative to HF-fed *Cd44*^+/+^ mice and were further decreased after PEGPH20 treatment (Figure 4a–c). *Cd44* deletion significantly decreased the phosphorylation of P38 and JNK MAPK, and this was more pronounced in *Cd44*^−/−^ mice when treated with PEGPH20 (Figure 4a,d). Furthermore, deletion of *Cd44* significantly reduced the protein expression of CD44 (Figure 4a,f) but had no effect on the protein expression of RHAMM (Figure 4a,g) in the kidney. ROCK2 and ERK/Akt phosphorylation was downregulated in HF-fed *Cd44*^−/−^ mice when compared with HF-fed *Cd44*^+/+^ mice, and PEGPH20 had no further effects (Figure 4a,h–j). mRNA expression of IL-1β, TNF-α, and IL-6 was markedly decreased, and IL-10 mRNA was increased in the HF-fed *Cd44*^−/−^ mice in comparison to the HF-fed *Cd44*^+/+^ mice. PEGPH20 treatment in *Cd44*^−/−^ mice further reduced IL-1β, TNF-α, and IL-6 mRNA expression and increased IL-10 mRNA expression compared to vehicle-treated *Cd44*^−/−^ mice (Figure 4k–n).

### 3.5. Global Hmmr Gene Deletion Reduced Obesity-Induced HA Accumulation, Glomerular Expansion, Tubular Damage, Renal Dysfunction, and Fibrosis

**Figure 5:**
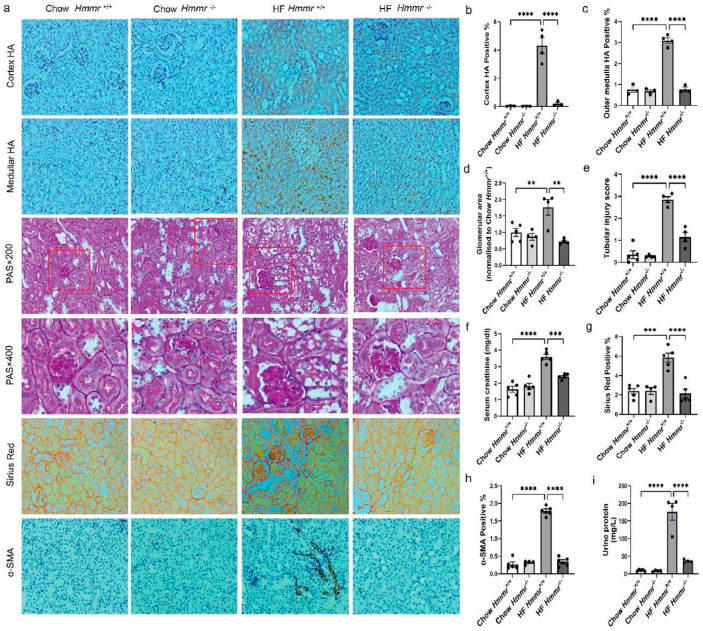
Global *Hmmr* gene deletion attenuated obesity-induced HA accumulation, glomerular area expansion, tubular damage, renal dysfunction, and fibrosis. (**a**) Images of HA, PAS, Sirius Red, and α-SMA staining in kidneys from *Hmmr*^+/+^ mice and *Hmmr*^−/−^ mice, both with either chow or high fat diet for 16 weeks. Representative images (×20 magnification) are shown for HA, Sirius Red, and α-SMA staining, and images (×20 and ×40 magnification) are shown for PAS staining. Areas of HA-positive (%), glomeruli, Sirius red-positive (%), and α-SMA-positive (%) were measured using the Image J program. (**b**,**c**) Quantification of HA content. (**d**) Glomerular area. (**e**) Tubular injury score. (**f**) Serum creatinine concentration. (**g**) Quantification of collagen deposition. (**h**) Quantification of α-SMA expression. (**i**) Urine albumin concentration. Data are presented as mean ± SEM. For all panels, one-way ANOVA followed by Tukey’s post-test for multiple comparisons was used for the analysis of statistical significance. Significance: ** *p* < 0.01, *** *p* < 0.001, **** *p* < 0.0001. Areas highlighted by red squares are further magnified to 400×.

The role of RHAMM (encoded by *Hmmr*) in renal injury in ORKP was next studied. The body weights of *Hmmr*^−/−^ and *Hmmr*^+/+^ mice were not different on either the chow or HF diet (Supplemental Figure S1a,b). The systolic and diastolic blood pressures were lower in chow-fed *Hmmr*^−/−^ mice than chow-fed *Hmmr*^+/+^ mice but were similar between genotypes on the HF diet (Supplemental Figure S1c,d). In the renal cortex and outer medulla, a similar level of HA was observed in chow-fed *Hmmr*^+/+^ and *Hmmr*^−/−^ mice. However, the HF diet significantly increased HA accumulation in *Hmmr*^+/+^ mice, but not in *Hmmr*^−/−^ mice (Figure 5a–c). Consistent with the results shown earlier, HF diet feeding in *Hmmr*^+/+^ mice increased the glomerular area, tubular injury score, serum creatinine concentration, collagen deposition, and α-SMA expression (Figure 5a,d–h). Moreover, HF diet feeding in *Hmmr*^+/+^ mice also resulted in albuminuria, another important marker of kidney dysfunction. (Figure 5i). *Hmmr* gene deletion significantly attenuated all these diet-induced effects.

### 3.6. Global Hmmr Gene Deletion Blocked the Activation of TGF-β1/Smad2/3, P38/JNK MAPK, and HA/CD44 Pathways and Prevented Inflammation in the Kidney of Obese Mice

**Figure 6:**
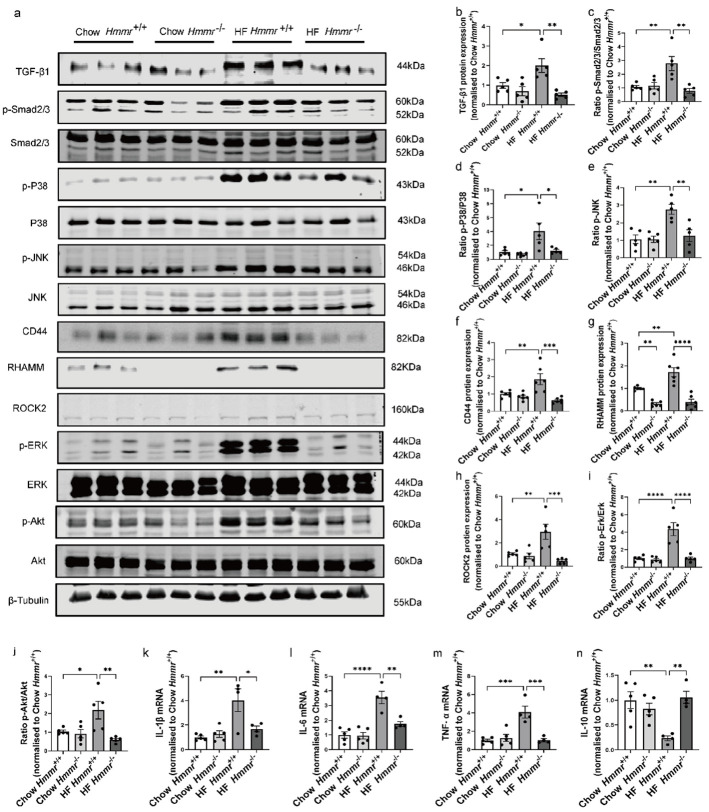
Global *Hmmr* gene deletion blocked the activation of TGF-β1/Smad2/3, P38/JNK MAPK, and HA/CD44 pathways and prevented obesity-induced inflammation. (**a**–**j**) Representative File S1 Western Blot images and quantification of protein expression of the TGF-β1/Smad2/3 pathway, P38/JNK MAPK pathway, and CD44/RHAMM and HA/CD44 pathway-associated proteins (ROCK2/Erk/Akt) in kidneys from *Hmmr*^+/+^ mice and *Hmmr*^−/−^ mice, both with either chow or high fat diet for 16 weeks. N = 5 male mice. (**k**–**n**) mRNA expression of IL-1β (**k**), IL-6 (**l**), TNF-α (**m**), and IL-10 (**n**) assessed by qRT–PCR. N = 4–5 male mice. Data are presented as mean ± SEM. For all panels, one-way ANOVA followed by Tukey’s post-test for multiple comparisons was used for the analysis of statistical significance. Significance: * *p* < 0.05, ** *p* < 0.01, *** *p* < 0.001, **** *p* < 0.0001.

As in the previous results, HF diet feeding increased the TGF-β1 expression, phosphorylation of Smad2/3, P38/JNK MAPK phosphorylation (Figure 6a–d), CD44 and RHAMM protein expression (Figure 6a,f,g), ROCK2 expression (Figure 6a,h), and Akt and ERK phosphorylation (Figure 6a,i,j) in the kidney of *Hmmr*^+/+^ mice. These diet-induced effects were prevented in HF-fed *Hmmr*^−/−^ mice. Furthermore, the mRNA expression of IL-1β, TNF-α, and IL-6 were increased, while the IL-10 mRNA was decreased in *Hmmr*^+/+^ mice by the HF diet. These diet-induced changes in cytokine expression were prevented in *Hmmr*^−/−^ mice (Figure 6k–n). However, the inflammasome NLRP3 mRNA was not affected either by the HF diet or RHAMM deletion (Supplemental Figure S2a).

To further examine whether the observed increase in CD44 and RHAMM expression in ORKP was due to the increased infiltration of immune cells in the kidney, we measured mRNA and protein expression of F4/80, a macrophage marker. Neither F4/80 mRNA nor protein expression was affected by HF diet feeding or genetic deletion of *Hmmr* (Supplemental Figure S2b–d), suggesting unchanged macrophage infiltration.

It is also interesting to note that the deletion of RHAMM prevented HF-induced CD44 elevation, yet the deletion of CD44 had no impact on RHAMM expression. These results suggest that RHAMM might be an upstream regulator of CD44. To test this hypothesis, we knocked down RHAMM in PT cells. Indeed, siRNA knockdown of RHAMM led to the decreased protein expression of CD44 and decreased phosphorylation of Akt (Supplemental Figure S3).

### 3.7. CD44 and RHAMM Were Upregulated in Insulin-Resistant Human Kidney Cells

**Figure 7:**
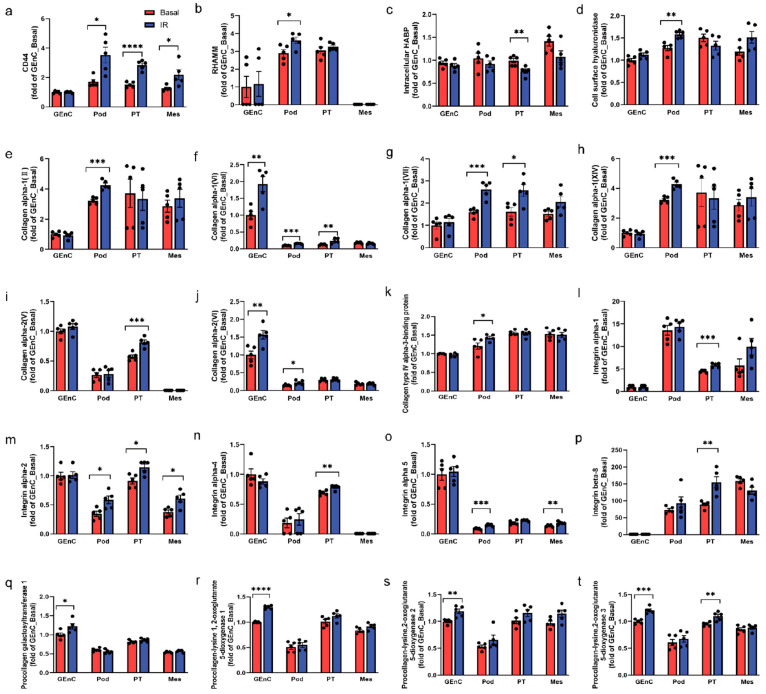
Proteomics analysis of protein expression in basal (Basal) vs. insulin-resistant (IR) glomerular endothelial cells (GEnC), podocytes (Pod), proximal tubular cells (PT), and mesangial cells (MES). (**a**) CD44; (**b**) RHAMM; (**c**) intracellular HA binding protein (HABP); (**d**) cell surface hyaluronidase; (**e**–**k**) protein expression of various collagen isoforms. Collagen alpha-1 (II) (**e**), collagen alpha-1 (VI) (**f**), collagen alpha-1 (VII) (**g**), collagen alpha-1 (XIV) (**h**), collagen alpha-2 (V) (**i**), collagen alpha-2 (VI) (**j**) and collagen type IV alpha-3-binding protein (**k**); (**l**–**p**) protein expression of collagen receptor integrins alpha-1 (**l**), integrins alpha-2 (**m**), integrins alpha-4 (**n**), integrins alpha-5 (**o**), and integrins beta-8 (**p**); (**q**–**t**) protein expression of collagen-modifying enzymes: procollagen galactosyltransferase 1 (**q**), procollagen-lysine 1, 2-oxoglutarate 5-dioxygenase 1 (**r**), procollagen-lysine,2-oxoglutarate 5-dioxygenase 2 (**s**), and procollagen-lysine,2-oxoglutarate 5-dioxygenase 3 (**t**). N = 5. Data are presented as mean ± SEM. For all panels, the unpaired two-tailed Student’s t test was used for the analysis of statistical significance between basal and insulin-resistant cells. Significance: * *p* < 0.05, ** *p* < 0.01, *** *p* < 0.001, **** *p* < 0.0001. Protein expression was normalised to the basal condition in GEnC and presented as fold changes.

We next investigated which cell types in the kidney are responsible for ORKP using conditionally immortalised human kidney cell lines [30]. Obesogenic and insulin resistant conditions were induced by incubating cells with high glucose, high insulin, and inflammatory cytokines [22]. Proteomic analysis revealed that CD44 protein expression was increased in insulin resistant podocytes, PT cells, and mesangial cells and not in GEnC (Figure 7a). Interestingly, RHAMM expression was only increased in insulin-resistant podocytes (Figure 7b). HA remodelling was observed in insulin-resistant podocytes and PT cells, as intracellular HA binding protein (HABP) was decreased in insulin-resistant PT cells, whereas cell surface hyaluronidase expression was increased in insulin-resistant podocytes (Figure 7c,d). Moreover, insulin resistance caused a considerable collagen accumulation in podocytes, PT, and GEnC cells as evidenced by the increased expression of various collagen isoforms, although not in mesangial cells (Figure 7e–k). Consistently, the protein expression of collagen receptor integrins was also increased by insulin resistance, mostly in PT cells, to a lesser extent in podocytes and mesangial cells, and not in GEnC (Figure 7l–p). Intriguingly, collagen remodelling (e.g., glycosylation and crosslinking) was induced by insulin resistance mainly in GEnC as evidenced by the increased expression of collagen-modifying enzymes (Figure 7q–t). These results provide insight into the role of different cell types of the kidney in obesity- and insulin resistance-associated renal injury.

### 3.8. Increased CD44 and RHAMM Expression Correlated with a Decline in Kidney Function in Humans

**Figure 8:**
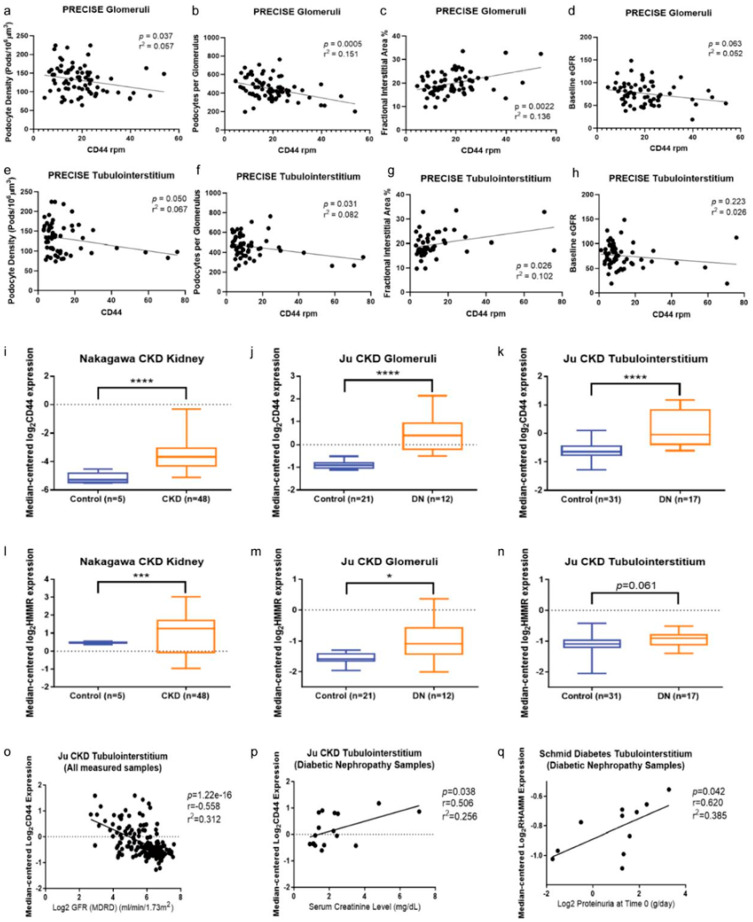
Increased CD44 and RHAMM expression correlated with a decline in kidney function in humans. (**a**–**h**) Correlations of CD44 gene expression in the glomeruli and tubulointerstitium with podocyte density (**a**,**e**), podocyte numbers per glomerulus (**b**,**f**), factional interstitial area (**c**,**g**), and baseline eGFR (**d**,**h**) in the PRECISE study [24]. (**i**–**n**) CD44 and RHAMM gene expression was analysed by Nephroseq v5 using datasets of Nakagawa CKD (chronic kidney disease) Kidney [27] and Ju CKD Glomeruli and Tubulointerstitium [28]. Two-tailed student’s t-tests were used for statistical comparison. (**o**–**q**) Correlations of CD44 and RHAMM gene expression with GFR, serum creatinine level, and proteinuria were analysed by Nephroseq v5 using datasets of Ju CKD Tubulointerstitium and Schmid Diabetes Tubulointerstitium [29]. Pearson correlations were performed for statistical analysis with *p* measuring statistical significance, r measuring the linear correlation between two variables, and r^2^ measuring how close the data are to the fitted regression line. Significance: * *p* < 0.05, *** *p* < 0.005, **** *p* < 0.001.

To test the human relevance of the mouse work, we analysed the gene expression of CD44 and RHAMM in the PRECISE study [24]. Fresh normal tissue from the unaffected part of a surgically removed kidney, with no pathological features, was analysed. This patient cohort had a mean baseline eGFR of 78 mL/min/1.73 m^2^. Our analysis showed that CD44 gene expression both in the glomeruli and tubulointerstitium was negatively correlated with the podocyte density and podocyte number per glomerulus (Figure 8a,b,e,f) and positively correlated with fractional interstitial area (Figure 8c,g). However, CD44 gene expression did not statistically correlate with the baseline eGFR, despite a trend for glomeruli CD44 (*p* = 0.063) (Figure 8d,h). This may be attributed to the generally high baseline eGFR in this patient cohort. The gene expression of RHAMM was low and not statistically correlated with any of these parameters in this patient cohort.

Furthermore, we analysed CD44 and RHAMM gene expression in patients with more advanced kidney diseases. Using publicly available transcriptomic datasets from the Nephroseq repository, we found that the gene expression of CD44 and RHAMM was increased in kidney biopsies of patients with CKD and diabetic nephropathy in comparison to those from healthy living donors (Figure 8i–n). Functionally, CD44 gene expression was negatively correlated with GFR and positively correlated with serum creatinine concentrations in patients with diabetic nephropathy (Figure 8o,p). Likewise, RHAMM expression was positively correlated with proteinuria in patients with diabetic nephropathy (Figure 8q).

## 4. Discussion

**Figure 9:**
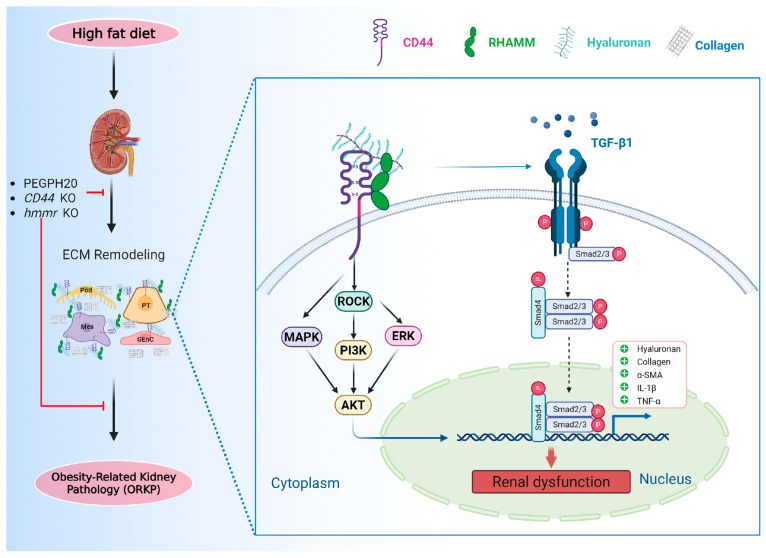
Working model for HA-CD44/RHAMM pathway in the pathogenesis of ORKP. Obesity induces renal ECM remodelling, including HA accumulation. HA binds to CD44 and RHAMM, activating TGF-β1/Smad2/3, P38/JNK MAPK, and ERK/Akt pathways, promoting transcription of profibrotic and proinflammatory genes, and contributing to the development of ORKP. Pharmacological and genetic disruptions of HA, CD44, or RHAMM prevents obesity-induced kidney damage.

In the present study, we identified a novel role for HA and its membrane receptors, CD44 and RHAMM, in the pathogenesis of ORKP. Our results suggest a working model where obesity induces renal HA accumulation and CD44 and RHAMM expression, which activates the TGF-β1/Smad2/3, P38/JNK MAPK, and ROCK2/ERK/Akt pathways, promoting transcription of profibrotic and proinflammatory genes and contributing to the development of ORKP. Pharmacological and genetic disruptions of HA, CD44, or RHAMM prevents obesity-induced kidney damage (Figure 9). Despite the previous implication of CD44 and RHAMM in renal fibrosis and disease, this study for the first time provides integral evidence for an important role of HA and both of its receptors in ORKP.

Upon kidney injury, profibrotic factors can be secreted by injured tubular epithelia and infiltrated inflammatory cells to promote fibroblast differentiation into myofibroblasts and ECM production, leading to renal fibrosis [31]. Tubulointerstitial fibrosis was observed in obesity-related kidney disease [32]. Renal fibrosis is characterised by pathological deposition of the ECM, which disrupts the normal kidney architecture and damages renal function [33]. The ECM HA was shown to be induced in the initial response phase of kidney injury and remodelled during the disease progression of AKI, CKD, and diabetic nephropathy [3], suggesting a pathological role of HA in kidney injury. Here, we also showed increased HA accumulation in ORKP in mice. Fibroblast differentiation into myofibroblasts is regulated by the classical TGF-β1-dependent Smad-signalling pathway, yet TGF-β1-mediated fibroblast differentiation was dependent upon HA and CD44 [34,35]. HA production by HA synthase-2 facilitated TGF-β1-dependent fibroblast differentiation via promoting CD44 interaction with epidermal growth factor receptor (EGFR) within membrane-bound lipid rafts [34]. Our results showing that the pharmacological reduction in HA by PEGPH20 or the genetic deletion of CD44 ameliorated renal fibrosis (i.e., decreased HA and collagen deposition) and reduced fibroblast differentiation (i.e., decreased α-SMA and TGF-β1/Smad2/3 signalling) in HF-fed mice were consistent with these previous findings [34].

In addition, CD44 has been demonstrated to be associated with the pathogenesis of crescentic glomerulonephritis [7], AKI [11], lupus nephritis [36,37] and CsA-induced renal injury [38]. CD44 was found to be upregulated and located in dilated tubules in the kidneys of a rat model of AKI [39]. Moreover, increased CD44 in glomerular parietal epithelial cells in aged mice contributed to glomerular hypertrophy and lower podocyte density, accompanied by segmental and global glomerulosclerosis [40]. In line with this, our results showed that HF-diet feeding for 16 weeks increased renal CD44 protein expression, and the genetic deletion of CD44 in mice ameliorated obesity-induced tubular injury and renal dysfunction, providing evidence for a crucial role of CD44 in ORKP.

The biological function of RHAMM is complex, and its extracellular and intracellular functions differ markedly. RHAMM plays a vital role in inflammation, angiogenesis [41,42], and a variety of tissue repair processes [43,44]. RHAMM interacts with ERK1/2 to regulate tumour metastasis [45] and is necessary for CD44-mediated skin wound healing [46]. RHAMM contributes to progressive fibrosis and is associated with systemic sclerosis [47]. However, the role of RHAMM in ORKP had not been previously studied. In the present study, we observed that HF-diet feeding in mice increased RHAMM protein expression, and *Hmmr* deletion in HF-fed mice significantly reduced renal fibrosis, decreased glomerular areas, and improved tubular injury and renal dysfunction. Our data suggest a vital role of RHAMM in ORKP for the first time.

The formation of a triple complex between HA, CD44, and RHAMM on the cell surface during tumorigenesis has been reported [48]. RHAMM enhances CD44 surface localisation, stabilises the HA-CD44 interaction, and contributes to the activation of ERK1/2 signalling [49]. It has been reported that cell surface RHAMM associates with several integral protein and non-protein tyrosine kinase receptors including TGF Receptor-1 [42], CD44 [46] and CD44-EGFR complexes [34,50], to regulate HA deposition and CD44 and RHAMM protein expression. In our study, HF diet-induced increases in CD44 protein expression were abolished in HF-fed *Hmmr*-null mice. We speculate that the beneficial action of *Hmmr* deletion was associated with the reduced cell surface HA-CD44-RHAMM complexes, but this needs to be further explored.

Obesity is a chronic low-grade inflammatory condition characterised by the up-regulation of pro-inflammatory cytokines (e.g., TNF-α, IL-6, IL-1β, MCP-1) and free fatty acids in the circulation, as well as the activation of inflammatory pathways (e.g., P38/JNK MAPK pathways), which contribute to kidney hypertrophy and dysfunction. Our results support this concept, where renal fibrosis and dysfunction were associated with elevated inflammation (i.e., increased expression of pro-inflammatory cytokines, decreased expression of anti-inflammatory cytokines, and decreased phosphorylation of P38/JNK). Conversely, in the mouse models tested in the present study, these inflammatory markers were reduced, associated with improved renal outcomes. Moreover, recent evidence highlights the NLRP3 inflammasome as a key mediator of chronic inflammation in obesity and insulin resistance. Nutrient excess activates the NLRP3 inflammasome-caspase 1 pathway, leading to the maturation of IL-1β and IL-18, key pro-inflammatory cytokines released by immune cells infiltrating adipose tissue in obese subjects [51]. The activation of the inflammasome and its target cytokines, IL-1β and IL-18, are important mediators in innate immunity and contribute to the development of obesity-induced inflammation and insulin resistance [52,53]. The fact that we observed an increase in IL-1β mRNA in ORKP, which was reversed when the HA-CD44/RHAMM pathway was inhibited, suggests a potential implication of the NLRP3 inflammasome pathway in ORKP. However, our preliminary data showed no changes in NLRP3 mRNA expression by HF diet feeding or RHAMM deletion. Therefore, future investigations are warranted for the involvement of the NLRP3 inflammasome in ORKP.

There is mounting evidence that ECM HA accumulation and increased CD44 expression are associated with obesity-related metabolic disorders, such as insulin resistance [54,55]. Early ECM remodelling is associated with obesity-associated insulin resistance in skeletal muscle, liver, and adipose tissue, where increased deposition of ECM components (e.g., collagens and HA) and their interaction with membrane bound receptors such as integrins and CD44 contribute to insulin resistance in obese mice [15,56,57,58]. The kidney also responds to the hormone insulin, and reduced insulin action in podocytes leads to impaired glomerular and renal function. In addition, insulin resistance prevails in patients with CKD and contributes to the progression of renal disease [59]. The relationship between insulin resistance and CKD also extends to ORKP. Patients with ORKP exhibited approximately 45% podocyte loss, and insulin resistance, specifically in podocytes, triggers podocyte morphology changes and apoptosis [60]. Here, we showed that in vitro obesogenic and insulin-resistant human kidney cells also underwent ECM remodelling, evidenced by changes in HA catabolism, increased deposition of collagens, changes in collagen modification, as well as increased expression of membrane ECM receptors including integrins, CD44, and RHAMM. Interestingly, these remodifications are cell type specific. While HA remodelling, collagen deposition, and ECM receptor upregulation primarily occurred in podocytes and PT cells, collagen modification and crosslinking occurred in GEnC cells. These results support our concept that ECM-receptor activation contributes to insulin resistance [15,56], as podocytes and PT cells are the main cell types responsive to insulin in the kidney [61]. However, the importance and relative contribution of each cell type to the pathological changes in ORKP are unknown and remain to be elucidated.

It is important to acknowledge that the in vitro model of insulin resistance used in this study mimics combined metabolic/inflammatory stress. Our findings, therefore, may not fully translate to the pathophysiological conditions of insulin resistance in vivo. The in vitro model has several advantages, including the ability to isolate specific cellular responses to insulin resistance and to control for confounding variables that are present in in vivo settings. This allows for a more detailed examination of the molecular mechanisms underlying ECM remodelling and insulin resistance, in a cell type-specific manner. However, the in vivo environment is more complex, involving multiple organ systems and regulatory mechanisms that are not fully captured in an in vitro setting. Therefore, while our in vitro model provides valuable insights into the cellular and molecular changes associated with insulin resistance, further studies in animal models and clinical settings are necessary to fully understand the pathophysiology of insulin resistance and its impact on ECM remodelling in the kidney.

It is worth noting that CD44 and RHAMM are also highly expressed in immune cells [62]. Therefore, it cannot be ruled out that elevated CD44 and RHAMM expression in the diseased kidneys reflect increased immune cell infiltration. However, our preliminary data showed that macrophage infiltration into the kidney was not affected either by HF diet feeding or RHAMM deletion in mice. Yet the infiltration of other immune cells was unknown. Single cell RNA sequencing, spatial transcriptomics and proteomics, and immunohistochemical detection of the cellular localisation of CD44 and RHAMM would be beneficial to ascertain the cell type-specific contribution of CD44 and RHAMM to the pathological changes in ORKP.

Although a recent study showed that ablation of CD44 protected mice from HF diet-induced obesity [63], the CD44-deficient mice used in our study exhibited no changes in obesity or adiposity [15]. Likewise, the RHAMM-deficient mice also showed no changes in body weight. These data suggest that the observed renal protective role of CD44 and RHAMM in our study was not due to a reduction in obesity. However, we have previously shown that treatment of PEGPH20 at 1 mg/kg body weight caused a small but significant reduction in body weight gain and fat mass gain, despite no changes in the absolute body weights of obese mice [14]. These results suggest that the observed renal benefits of PEGPH20 may not be solely attributable to the direct effects of PEGPH20 on HA metabolism in the kidney. The systemic metabolic changes induced by PEGPH20, such as reduced adiposity, could also play a significant role in these improvements. Similarly, 4-methylumbelliferone (4-MU), an alternative and more effective inhibitor of HA synthesis, has been shown to ameliorate hypertriglyceridaemia and hyperglycaemia [64]. This effect is partly mediated by modulating hepatic lipid metabolism and the antioxidant defence system, as well as by increasing adiponectin concentrations [64]. Therefore, it remains unclear whether the renal benefits of HA reduction are primarily due to the direct modulation of HA in the kidney or are influenced by broader systemic changes.

Lastly, it is worth noting that blood pressure influences kidney function by regulating blood flow to the kidneys, thereby controlling their sodium and water excretion to maintain blood pressure. Although we measured systolic and diastolic pressures in the RHAMM deficient mice, blood pressures in mice treated with PEGPH20 or deficient of CD44 have not been measured or reported previously. Therefore, the potential impact of blood pressure changes on their renal function of these models is uncertain and will merit future investigations.

## 5. Conclusions

This study demonstrates that HA and its membrane receptors, CD44 and RHAMM, mediate ORKP, possibly via activation of the TGF-β1/Smad2/3, P38/JNK MAPK, and ROCK/ERK pathways. We further provide evidence that pharmacological and genetic ablation of these molecules in mice reverses the adverse renal effects of obesity; therefore, an intervention targeting the HA-CD44/RHAMM pathway may represent a novel therapeutic strategy against the progression of obesity-induced kidney injury. Mechanistic work in insulin-resistant human kidney cells in vitro illustrates an association between renal ECM remodelling and insulin resistance. Further studies to elucidate the causal relationship between renal ECM remodelling, kidney insulin resistance, and subsequent ORKP are warranted. Lastly, we show the clinical relevance of CD44 and RHAMM in human CKD and diabetic nephropathy, highlighting their implications and therapeutic potential in wider human kidney diseases.

## Data Availability

All data are included in the manuscript and/or Supplemental Materials. Original data will be made available upon requests after publication.

## Supplementary Materials

The following supporting information can be downloaded at: https://www.mdpi.com/article/10.3390/biom15111598/s1. Table S1. Sequences of primers used for real-time RT-PCR. Figure S1. Effects of global Hmmr gene deletion on body weight, systolic, diastolic and pulse blood pressures on chow and HF diet. Figure S2. Effects of global Hmmr gene deletion on macrophage infiltration and inflammasome activation on chow and HF diet. Figure S3. The effect of RHAMM knockdown on CD44 expression and Akt signaling in proximal tubular (PT) cells. File S1. Western Blot images.

## References

[1] Dogantekin E., Akgul T., Eser E.P., Kotanoglu M., Bayburtluoglu V., Hucumenoglu S. (2022). The effect of intraurethral hyaluronic acid on healing and fibrosis in rats with experimentally induced urethral trauma. Int. Urol. Nephrol..

[2] Stridh S., Palm F., Hansell P. (2012). Renal interstitial hyaluronan: Functional aspects during normal and pathological conditions. Am. J. Physiol. Regul. Integr. Comp. Physiol..

[3] Kaul A., Singampalli K.L., Parikh U.M., Yu L., Keswani S.G., Wang X. (2022). Hyaluronan, a double-edged sword in kidney diseases. Pediatr. Nephrol..

[4] Weng X., Maxwell-Warburton S., Hasib A., Ma L., Kang L. (2022). The membrane receptor CD44: Novel insights into metabolism. Trends Endocrinol. Metab..

[5] Decleves A.E., Caron N., Nonclercq D., Legrand A., Toubeau G., Kramp R., Flamion B. (2006). Dynamics of hyaluronan, CD44, and inflammatory cells in the rat kidney after ischemia/reperfusion injury. Int. J. Mol. Med..

[6] Bao Y., Wu W., Lin J., Yang Y., Lin S., Su J., Qin Y., Wang B., Duan S. (2025). Increased HA/CD44/TGFβ signaling implicates in renal fibrosis of a Col4a5 mutant Alport mice. Mol. Med..

[7] Eymael J., Sharma S., Loeven M.A., Wetzels J.F., Mooren F., Florquin S., Deegens J.K., Willemsen B.K., Sharma V., van Kuppevelt T.H. (2018). CD44 is required for the pathogenesis of experimental crescentic glomerulonephritis and collapsing focal segmental glomerulosclerosis. Kidney Int..

[8] Savani R.C., Wang C., Yang B., Zhang S., Kinsella M.G., Wight T.N., Stern R., Nance D.M., Turley E.A. (1995). Migration of bovine aortic smooth muscle cells after wounding injury. The role of hyaluronan and RHAMM. J. Clin. Investig..

[9] Jaskula K., Sacharczuk M., Gaciong Z., Skiba D.S. (2021). Cardiovascular Effects Mediated by HMMR and CD44. Mediat. Inflamm..

[10] Misra S., Hascall V.C., Markwald R.R., Ghatak S. (2015). Interactions between Hyaluronan and Its Receptors (CD44, RHAMM) Regulate the Activities of Inflammation and Cancer. Front. Immunol..

[11] Chen T.H., Liu C.T., Cheng C.Y., Sue Y.M., Huang N.J., Chen C.H. (2022). Oligosaccharides Ameliorate Acute Kidney Injury by Alleviating Cluster of Differentiation 44-Mediated Immune Responses in Renal Tubular Cells. Nutrients.

[12] Chi A., Shirodkar S.P., Escudero D.O., Ekwenna O.O., Yates T.J., Ayyathurai R., Garcia-Roig M., Gahan J.C., Manoharan M., Bird V.G. (2012). Molecular characterization of kidney cancer: Association of hyaluronic acid family with histological subtypes and metastasis. Cancer.

[13] Qi B., Lou Y., Zhu Y., Chen Y., Yang S., Meng F., Pan Z., Liu S., Yan G., Lu X. (2024). Elevated RHAMM as a biomarker for predicting diabetic kidney disease in patients with type 2 diabetes. Clin. Kidney J..

[14] Kang L., Lantier L., Kennedy A., Bonner J.S., Mayes W.H., Bracy D.P., Bookbinder L.H., Hasty A.H., Thompson C.B., Wasserman D.H. (2013). Hyaluronan accumulates with high-fat feeding and contributes to insulin resistance. Diabetes.

[15] Hasib A., Hennayake C.K., Bracy D.P., Bugler-Lamb A.R., Lantier L., Khan F., Ashford M.L.J., McCrimmon R.J., Wasserman D.H., Kang L. (2019). CD44 contributes to hyaluronan-mediated insulin resistance in skeletal muscle of high-fat-fed C57BL/6 mice. Am. J. Physiol. Endocrinol. Metab..

[16] Tolg C., Poon R., Fodde R., Turley E.A., Alman B.A. (2003). Genetic deletion of receptor for hyaluronan-mediated motility (Rhamm) attenuates the formation of aggressive fibromatosis (desmoid tumor). Oncogene.

[17] Hauet T., Mothes D., Goujon J.M., Caritez J.C., Carretier M., le Moyec L., Eugene M., Tillement J.P. (1997). Trimetazidine prevents renal injury in the isolated perfused pig kidney exposed to prolonged cold ischemia. Transplantation.

[18] Saleem M.A., O’Hare M.J., Reiser J., Coward R.J., Inward C.D., Farren T., Xing C.Y., Ni L., Mathieson P.W., Mundel P. (2002). A conditionally immortalized human podocyte cell line demonstrating nephrin and podocin expression. J. Am. Soc. Nephrol..

[19] Sarrab R.M., Lennon R., Ni L., Wherlock M.D., Welsh G.I., Saleem M.A. (2011). Establishment of conditionally immortalized human glomerular mesangial cells in culture, with unique migratory properties. Am. J. Physiol. Ren. Physiol..

[20] Wilmer M.J., Saleem M.A., Masereeuw R., Ni L., van der Velden T.J., Russel F.G., Mathieson P.W., Monnens L.A., van den Heuvel L.P., Levtchenko E.N. (2010). Novel conditionally immortalized human proximal tubule cell line expressing functional influx and efflux transporters. Cell Tissue Res..

[21] Satchell S.C., Tasman C.H., Singh A., Ni L., Geelen J., von Ruhland C.J., O’Hare M.J., Saleem M.A., van den Heuvel L.P., Mathieson P.W. (2006). Conditionally immortalized human glomerular endothelial cells expressing fenestrations in response to VEGF. Kidney Int..

[22] Lay A.C., Hurcombe J.A., Betin V.M.S., Barrington F., Rollason R., Ni L., Gillam L., Pearson G.M.E., Ostergaard M.V., Hamidi H. (2017). Prolonged exposure of mouse and human podocytes to insulin induces insulin resistance through lysosomal and proteasomal degradation of the insulin receptor. Diabetologia.

[23] Lay A.C., Tran V.D.T., Nair V., Betin V., Hurcombe J.A., Barrington A.F., Pope R.J., Burdet F., Mehl F., Kryvokhyzha D. (2024). Profiling of insulin-resistant kidney models and human biopsies reveals common and cell-type-specific mechanisms underpinning Diabetic Kidney Disease. Nat. Commun..

[24] Schaub J.A., O’Connor C.L., Shi J., Wiggins R.C., Shedden K., Hodgin J.B., Bitzer M. (2022). Quantitative morphometrics reveals glomerular changes in patients with infrequent segmentally sclerosed glomeruli. J. Clin. Pathol..

[25] Li H., Eksi R., Yi D., Godfrey B., Mathew L.R., O’Connor C.L., Bitzer M., Kretzler M., Menon R., Guan Y. (2022). Micro-dissection and integration of long and short reads to create a robust catalog of kidney compartment-specific isoforms. PLoS Comput. Biol..

[26] Pippin J.W., Kaverina N., Wang Y., Eng D.G., Zeng Y., Tran U., Loretz C.J., Chang A., Akilesh S., Poudel C. (2022). Upregulated PD-1 signaling antagonizes glomerular health in aged kidneys and disease. J. Clin. Investig..

[27] Nakagawa S., Nishihara K., Miyata H., Shinke H., Tomita E., Kajiwara M., Matsubara T., Iehara N., Igarashi Y., Yamada H. (2015). Molecular Markers of Tubulointerstitial Fibrosis and Tubular Cell Damage in Patients with Chronic Kidney Disease. PLoS ONE.

[28] Ju W., Nair V., Smith S., Zhu L., Shedden K., Song P.X.K., Mariani L.H., Eichinger F.H., Berthier C.C., Randolph A. (2015). Tissue transcriptome-driven identification of epidermal growth factor as a chronic kidney disease biomarker. Sci. Transl. Med..

[29] Schmid H., Boucherot A., Yasuda Y., Henger A., Brunner B., Eichinger F., Nitsche A., Kiss E., Bleich M., Gröne H.J. (2006). Modular activation of nuclear factor-kappaB transcriptional programs in human diabetic nephropathy. Diabetes.

[30] Balzer M.S., Rohacs T., Susztak K. (2022). How Many Cell Types Are in the Kidney and What Do They Do?. Annu. Rev. Physiol..

[31] Yuan Q., Tan R.J., Liu Y. (2019). Myofibroblast in Kidney Fibrosis: Origin, Activation, and Regulation. Adv. Exp. Med. Biol..

[32] Kim D.H., Chun S.Y., Lee E., Kim B., Yoon B., Gil H., Han M.H., Ha Y.S., Lee J.N., Kwon T.G. (2021). IL-10 Deficiency Aggravates Renal Inflammation, Fibrosis and Functional Failure in High-Fat Dieted Obese Mice. Tissue Eng. Regen. Med..

[33] Bülow R.D., Boor P. (2019). Extracellular Matrix in Kidney Fibrosis: More Than Just a Scaffold. J. Histochem. Cytochem. Off. J. Histochem. Soc..

[34] Midgley A.C., Rogers M., Hallett M.B., Clayton A., Bowen T., Phillips A.O., Steadman R. (2013). Transforming growth factor-β1 (TGF-β1)-stimulated fibroblast to myofibroblast differentiation is mediated by hyaluronan (HA)-facilitated epidermal growth factor receptor (EGFR) and CD44 co-localization in lipid rafts. J. Biol. Chem..

[35] Bourguignon L.Y., Singleton P.A., Zhu H., Zhou B. (2002). Hyaluronan promotes signaling interaction between CD44 and the transforming growth factor beta receptor I in metastatic breast tumor cells. J. Biol. Chem..

[36] Yung S., Chan T.M. (2012). The Role of Hyaluronan and CD44 in the Pathogenesis of Lupus Nephritis. Autoimmune Dis..

[37] Kadoya H., Yu N., Schiessl I.M., Riquier-Brison A., Gyarmati G., Desposito D., Kidokoro K., Butler M.J., Jacob C.O., Peti-Peterdi J. (2020). Essential role and therapeutic targeting of the glomerular endothelial glycocalyx in lupus nephritis. JCI Insight.

[38] Han D.H., Song H.K., Lee S.Y., Song J.H., Piao S.G., Yoon H.E., Ghee J.Y., Yoon H.J., Kim J., Yang C.W. (2010). Upregulation of hyaluronan and its binding receptors in an experimental model of chronic cyclosporine nephropathy. Nephrology.

[39] Matsushita K., Toyoda T., Yamada T., Morikawa T., Ogawa K. (2021). Specific expression of survivin, SOX9, and CD44 in renal tubules in adaptive and maladaptive repair processes after acute kidney injury in rats. J. Appl. Toxicol..

[40] Hamatani H., Eng D.G., Hiromura K., Pippin J.W., Shankland S.J. (2020). CD44 impacts glomerular parietal epithelial cell changes in the aged mouse kidney. Physiol. Rep..

[41] Hauser-Kawaguchi A., Luyt L.G., Turley E. (2019). Design of peptide mimetics to block pro-inflammatory functions of HA fragments. Matrix Biol..

[42] Park D., Kim Y., Kim H., Kim K., Lee Y.S., Choe J., Hahn J.H., Lee H., Jeon J., Choi C. (2012). Hyaluronic acid promotes angiogenesis by inducing RHAMM-TGFbeta receptor interaction via CD44-PKCdelta. Mol. Cells.

[43] Ma X., Pearce J.D., Wilson D.B., English W.P., Edwards M.S., Geary R.L. (2014). Loss of the hyaluronan receptor RHAMM prevents constrictive artery wall remodeling. J. Vasc. Surg..

[44] Tolg C., McCarthy J.B., Yazdani A., Turley E.A. (2014). Hyaluronan and RHAMM in wound repair and the “cancerization” of stromal tissues. Biomed. Res. Int..

[45] Gao C., Liu S., Wang Y., Cha G., Xu X. (2019). Effect of receptor for hyaluronan-mediated motility inhibition on radiosensitivity of lung adenocarcinoma A549 cells. Transl. Cancer Res..

[46] Tolg C., Hamilton S.R., Nakrieko K.A., Kooshesh F., Walton P., McCarthy J.B., Bissell M.J., Turley E.A. (2006). Rhamm^−/−^ fibroblasts are defective in CD44-mediated ERK1,2 motogenic signaling, leading to defective skin wound repair. J. Cell Biol..

[47] Wu K.Y., Kim S., Liu V.M., Sabino A., Minkhorst K., Yazdani A., Turley E.A. (2021). Function-Blocking RHAMM Peptides Attenuate Fibrosis and Promote Antifibrotic Adipokines in a Bleomycin-Induced Murine Model of Systemic Sclerosis. J. Investig. Dermatol..

[48] Carvalho A.M., Soares da Costa D., Paulo P.M.R., Reis R.L., Pashkuleva I. (2021). Co-localization and crosstalk between CD44 and RHAMM depend on hyaluronan presentation. Acta Biomater..

[49] Hamilton S.R., Fard S.F., Paiwand F.F., Tolg C., Veiseh M., Wang C., McCarthy J.B., Bissell M.J., Koropatnick J., Turley E.A. (2007). The hyaluronan receptors CD44 and Rhamm (CD168) form complexes with ERK1,2 that sustain high basal motility in breast cancer cells. J. Biol. Chem..

[50] Hatano H., Shigeishi H., Kudo Y., Higashikawa K., Tobiume K., Takata T., Kamata N. (2011). RHAMM/ERK interaction induces proliferative activities of cementifying fibroma cells through a mechanism based on the CD44-EGFR. Lab. Investig..

[51] Rheinheimer J., de Souza B.M., Cardoso N.S., Bauer A.C., Crispim D. (2017). Current role of the NLRP3 inflammasome on obesity and insulin resistance: A systematic review. Metab. Clin. Exp..

[52] Lee B.C., Lee J. (2014). Cellular and molecular players in adipose tissue inflammation in the development of obesity-induced insulin resistance. Biochim. Biophys. Acta.

[53] Engin A. (2024). Reappraisal of Adipose Tissue Inflammation in Obesity. Adv. Exp. Med. Biol..

[54] Nagy N., Czepiel K.S., Kaber G., Stefanovski D., Hargil A., Pennetzdorfer N., Targ R., Reghupaty S.C., Wight T.N., Vernon R.B. (2024). Hymecromone Promotes Longevity and Insulin Sensitivity in Mice. Cells.

[55] Kang H.S., Liao G., DeGraff L.M., Gerrish K., Bortner C.D., Garantziotis S., Jetten A.M. (2013). CD44 plays a critical role in regulating diet-induced adipose inflammation, hepatic steatosis, and insulin resistance. PLoS ONE.

[56] Kang L., Ayala J.E., Lee-Young R.S., Zhang Z., James F.D., Neufer P.D., Pozzi A., Zutter M.M., Wasserman D.H. (2011). Diet-induced muscle insulin resistance is associated with extracellular matrix remodeling and interaction with integrin alpha2beta1 in mice. Diabetes.

[57] Williams A.S., Kang L., Zheng J., Grueter C., Bracy D.P., James F.D., Pozzi A., Wasserman D.H. (2015). Integrin alpha1-null mice exhibit improved fatty liver when fed a high fat diet despite severe hepatic insulin resistance. J. Biol. Chem..

[58] Bugler-Lamb A.R., Hasib A., Weng X., Hennayake C.K., Lin C., McCrimmon R.J., Stimson R.H., Ashford M.L.J., Wasserman D.H., Kang L. (2021). Adipocyte integrin-linked kinase plays a key role in the development of diet-induced adipose insulin resistance in male mice. Mol. Metab..

[59] Spoto B., Pisano A., Zoccali C. (2016). Insulin resistance in chronic kidney disease: A systematic review. Am. J. Physiol. Renal Physiol..

[60] Yang S., Cao C., Deng T., Zhou Z. (2020). Obesity-Related Glomerulopathy: A Latent Change in Obesity Requiring More Attention. Kidney Blood Press. Res..

[61] Artunc F., Schleicher E., Weigert C., Fritsche A., Stefan N., Haring H.U. (2016). The impact of insulin resistance on the kidney and vasculature. Nat. Rev. Nephrol..

[62] Cui Z., Liao J., Cheong N., Longoria C., Cao G., DeLisser H.M., Savani R.C. (2019). The Receptor for Hyaluronan-Mediated Motility (CD168) promotes inflammation and fibrosis after acute lung injury. Matrix Biol..

[63] Wu Y., Ma J., Chen J., Liu X., Wang Z., Luo L., Sun C. (2025). Ablation of CD44 Attenuates Adipogenesis in White Adipocytes via the Tryptophan 5-Hydroxylase 2/5-Hydroxytryptamine Axis to Protect Mice from High-Fat Diet-Induced Obesity. Am. J. Pathol..

[64] Sim M.O., Ham J.R., Lee H.I., Seo K.I., Lee M.K. (2014). Long-term supplementation of umbelliferone and 4-methylumbelliferone alleviates high-fat diet induced hypertriglyceridemia and hyperglycemia in mice. Chem.-Biol. Interact..

