# Supplementary material for: A Novel Role of Hyaluronan and Its Membrane Receptors, CD44 and RHAMM, in Obesity-Related Kidney Pathology
Source: Biomolecules. 2025 Nov 14;15(11):1598. doi: 10.3390/biom15111598 (PMC12650540; doi:10.3390/biom15111598)
Supplement: Supplementary file 1 [file biomolecules-15-01598-s001.zip › biomolecules-3861081-supplementary.pdf]

TNF-α	Forward	TGCCACCTTTTGACAGTGAT
	Reverse	GATTTGAAGCTGGATGCTCT
IL-1β	Forward	TGCCACCTTTTGACAGTGAT
	Reverse	GATTTGAAGCTGGATGCTCT
IL-10	Forward	CTTACTGACTGGCATGAGGATCA
	Reverse	GCAGCTCTAGGAGCATGTGG
IL-6	Forward	GAAAAGAGTTGTGCAATGGCAAT
	Reverse	TTGGTAGCATCCATCATTTCTTTG
NLRP3		m00840904_m1 (Taqman^{TM})
ADGRE1		Mm00802529_m1 (Taqman^{TM})
18S		Mm04277571_s1 (Taqman^{TM})

Supplemental Table S1. Sequences of primers used for real-time RT-PCR.

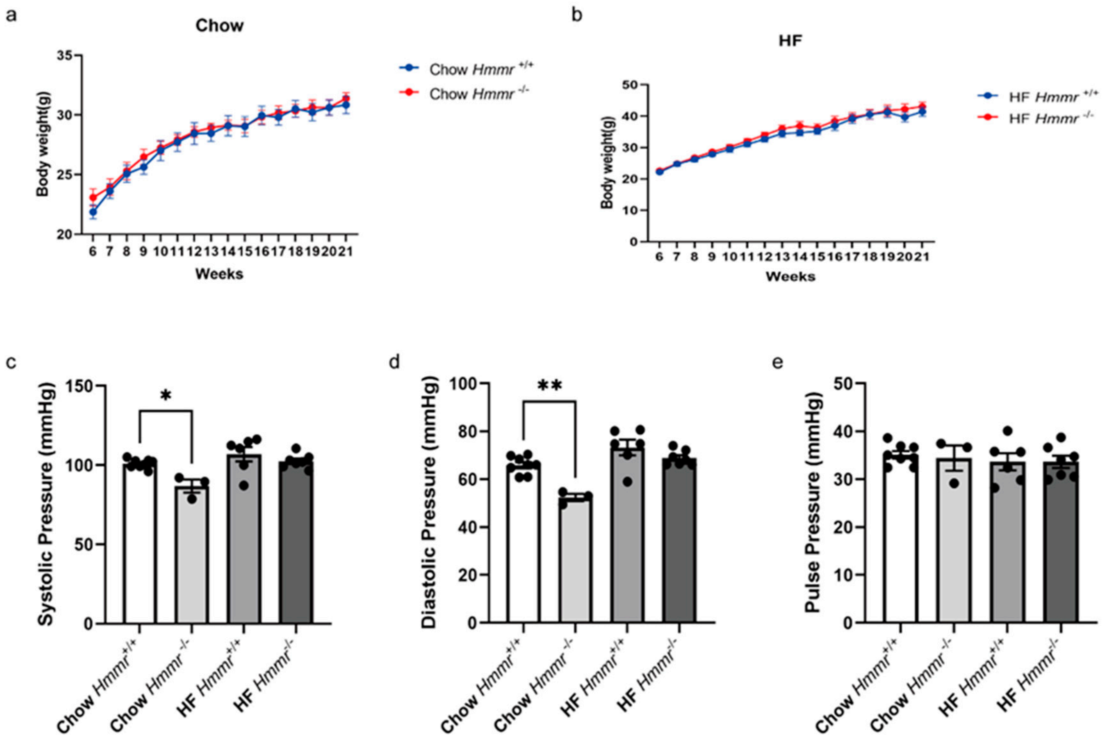

Supplemental Fig S1. Effects of global *Hmmr* gene deletion on body weight, systolic, diastolic and pulse blood pressures on chow and HF diet. a-b Body weights of global *Hmmr* gene deficient mice were monitored weekly, up to 21 wks on chow or HF diet. N = 6-7 male mice. **c-e** Systolic, diastolic and pulse blood pressures were monitored using a pressure volume (PV) loop analysis, N = 3-8 for chow and N = 6-7 for HF male mice. PV loop is considered a gold standard for the measurement of cardiac function. Mice were anaesthetized using 2% isoflurane (volume/volume). Admittance catheter (1.2F, Transonic) coupled to ADV500 data acquisition system (Transonic) visualised by LabChart (ADInstruments) sensors, was introduced into the aorta via the carotid artery to measure arterial pressure. During the procedure body temperature of mice was monitored by a rectal thermometer probe. The blood pressure data obtained from the experimental mice were analysed using Lab Chart Pro 8 software (ADInstruments). One-way ANOVA followed by Tukey's post-hoc test for multiple comparisons was used for the analysis of statistical significance. Significance *$p < 0.05$, **$p < 0.01$.

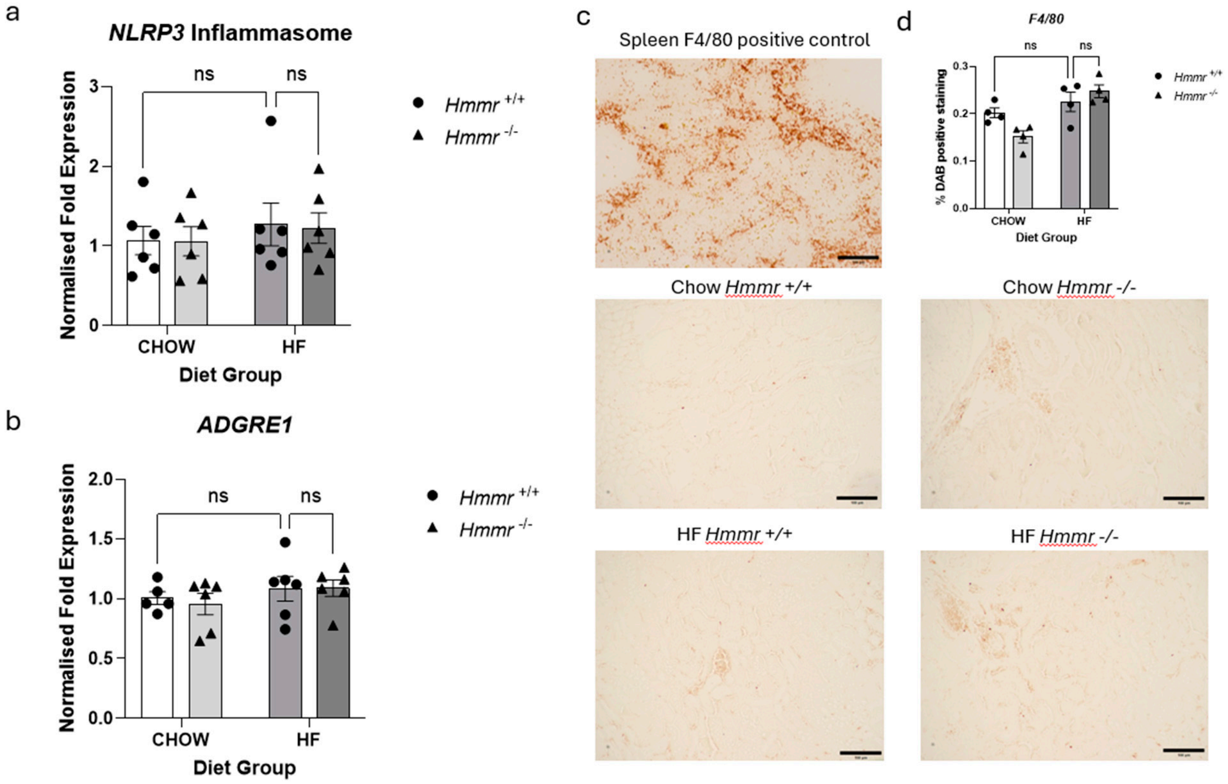

Supplemental Fig S2. Effects of global *Hmmr* gene deletion on macrophage infiltration and inflammasome activation on chow and HF diet. a-b qPCR quantification of mRNA expression of *NLRP3* and *ADGRE1 (encodes F4/80)*, normalized to chow *Hmmr*$^{+/+}$ group. **c** Representative image of F4/80 immunohistochemistry in renal tissues in the medulla and cortex. Paraffin-embedded kidney tissues were sectioned at 5µm and mounted onto staining slides. Expression of F4/80 was assessed by avidin biotin complex (with diaminobenzidine staining (#ab64264, ABCAM). Primary, secondary and HRP-Streptavadin were used at a 1/100 concentration and diaminobenzidine at a working concentration as per manufacturer. Images of the renal cortex and medulla were captured by SpotImaging microscope and quantified using ImageJ software. Image analysis was performed with consistent thresholds and under blinded conditions. **d** Expression of F4/80 protein were analysed using 3,3′-Diaminobenzidine immunohistochemistry staining, quantified by Image J. Statistical analysis was performed by two-way ANOVA. Significance *$p < 0.05$, **$p < 0.01$. ns: not significant.

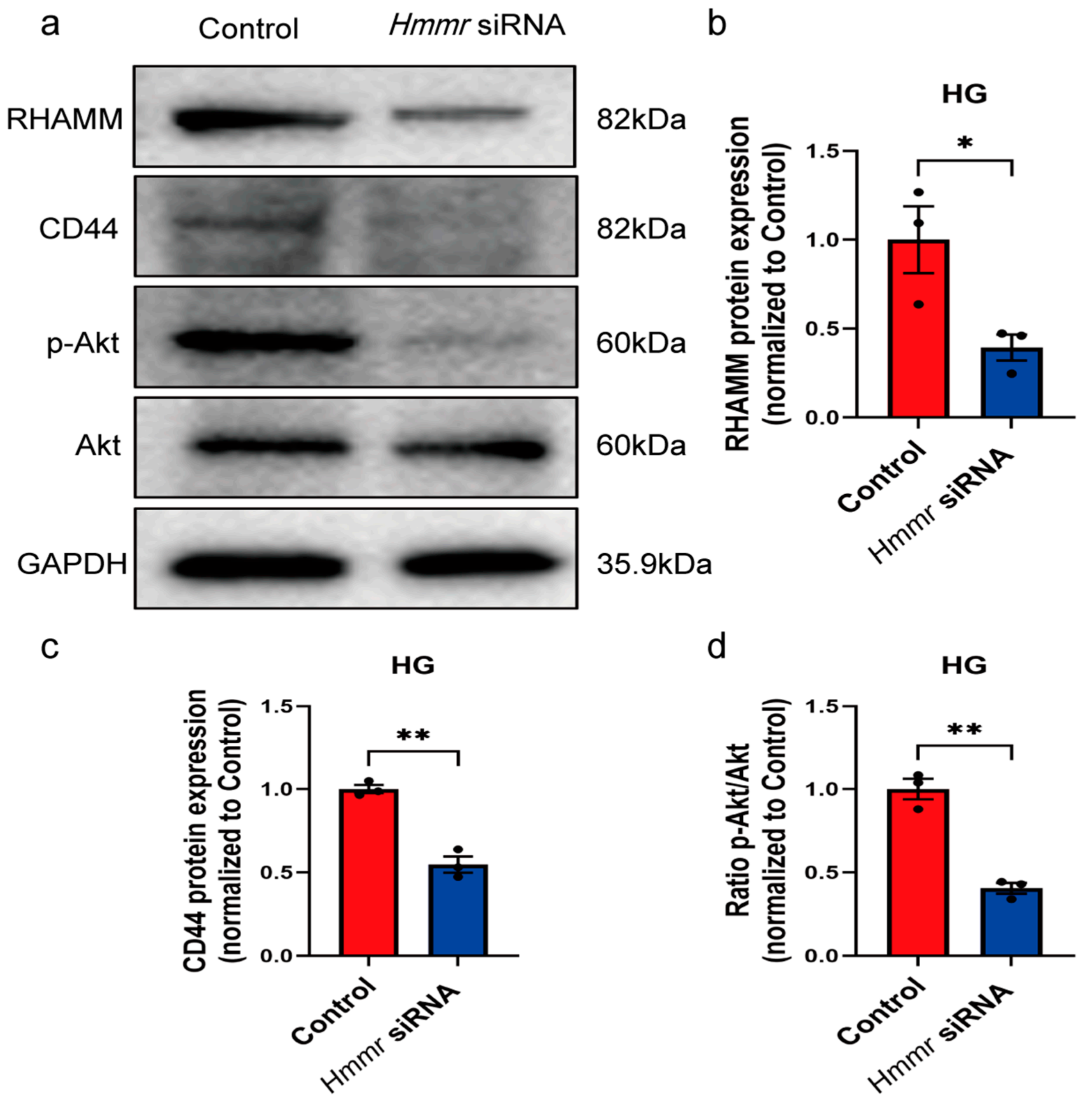

Supplemental Fig S3. The effect of RHAMM knockdown on CD44 expression and Akt signaling in proximal tubular (PT) cells. PT cells were treated with 25mmol·L^{-1} glucose and 40nmol·L^{-1} *Hmmr*-specific siRNA (Gene Adv), with 40nmol·L^{-1} scramble siRNA as a control. Representative western blot images and quantification of protein expression of the RHAMM, CD44 and Akt signaling in PT cells. N = 3. The unpaired, two-tailed Student's t test was used for the analysis of statistical significance. Significance *$p < 0.05$, **$p < 0.01$. HG: high glucose.
